# Incidence trends of airflow obstruction among European adults without asthma: a 20-year cohort study

**DOI:** 10.1038/s41598-020-60478-5

**Published:** 2020-02-26

**Authors:** Simone Accordini, Lucia Calciano, Alessandro Marcon, Giancarlo Pesce, Josep M. Antó, Anna B. Beckmeyer-Borowko, Anne-Elie Carsin, Angelo G. Corsico, Medea Imboden, Christer Janson, Dirk Keidel, Francesca Locatelli, Cecilie Svanes, Peter G. J. Burney, Deborah Jarvis, Nicole M. Probst-Hensch, Cosetta Minelli

**Affiliations:** 1 Unit of Epidemiology and Medical Statistics, Department of Diagnostics and Public Health, University of Verona, Verona, Italy; 2 Sorbonne Universités, INSERM UMR-S 1136, IPLESP, Team EPAR, F75012 Paris, France; 3 ISGlobal, Centre for Research in Environmental Epidemiology (CREAL), Barcelona, Spain; 4 Hospital del Mar Medical Research Institute (IMIM), Barcelona, Spain; 5 CIBER Epidemiología y Salud Pública (CIBERESP), Barcelona, Spain; 6 Universitat Pompeu Fabra (UPF), Barcelona, Spain; 7 Department of Epidemiology and Public Health, Swiss Tropical and Public Health Institute, Basel, Switzerland; 8 University of Basel, Basel, Switzerland; 9 Division of Respiratory Diseases, IRCCS ‘San Matteo’ Hospital Foundation-University of Pavia, Pavia, Italy; 10 Department of Medical Sciences: Respiratory, Allergy and Sleep Research, Uppsala University, Uppsala, Sweden; 11 Centre for International Health, Department of Global Public Health and Primary Care, University of Bergen, Bergen, Norway; 12 Department of Occupational Medicine, Haukeland University Hospital, Bergen, Norway; 13 Population Health and Occupational Disease, National Heart and Lung Institute, Imperial College London, London, UK; 14 MRC-PHE Centre for Environment and Health, Imperial College London, London, UK

**Keywords:** Chronic obstructive pulmonary disease, Risk factors

## Abstract

Investigating COPD trends may help healthcare providers to forecast future disease burden. We estimated sex- and smoking-specific incidence trends of pre-bronchodilator airflow obstruction (AO) among adults without asthma from 11 European countries within a 20-year follow-up (ECRHS and SAPALDIA cohorts). We also quantified the extent of misclassification in the definition based on pre-bronchodilator spirometry (using post-bronchodilator measurements from a subsample of subjects) and we used this information to estimate the incidence of post-bronchodilator AO (AO_post-BD_), which is the primary characteristic of COPD. AO incidence was 4.4 (95% CI: 3.5–5.3) male and 3.8 (3.1–4.6) female cases/1,000/year. Among ever smokers (median pack-years: 20, males; 12, females), AO incidence significantly increased with ageing in men only [incidence rate ratio (IRR), 1-year increase: 1.05 (1.03–1.07)]. A strong exposure-response relationship with smoking was found both in males [IRR, 1-pack-year increase: 1.03 (1.02–1.04)] and females [1.03 (1.02–1.05)]. The positive predictive value of AO for AO_post-BD_ was 59.1% (52.0–66.2%) in men and 42.6% (35.1–50.1%) in women. AO_post-BD_ incidence was 2.6 (1.7–3.4) male and 1.6 (1.0–2.2) female cases/1,000/year. AO incidence was considerable in Europe and the sex-specific ageing-related increase among ever smokers was strongly related to cumulative tobacco exposure. AO_post-BD_ incidence is expected to be half of AO incidence.

## Introduction

Chronic Obstructive Pulmonary Disease (COPD) is a major cause of chronic morbidity and mortality worldwide^1^, and it represents an important public health challenge, being both a preventable and treatable disease^2^. Globally, the COPD burden is projected to increase in coming decades because of continued exposure to risk factors and ageing of the population^3^.

COPD is characterised by a progressive airflow obstruction that is not fully reversible^4^. According to the Global Initiative for Chronic Obstructive Lung Disease guidelines^2^, the diagnosis of COPD requires post-bronchodilator spirometry, and the use of pre-bronchodilator measurements may lead to some misclassification of the disease^5^. However, post-bronchodilator spirometry has been adopted in epidemiological studies in recent years only.

Investigating how COPD rates could be affected by changes in major risk factors may help healthcare providers and decision makers to forecast the disease burden and to optimise clinical and public health strategies. In particular, tobacco smoking is the main preventable cause of COPD and it is still the most important health hazard in Europe^6^. Epidemiological tools for Health Impact Assessment^7^ are available to forecast changes in the COPD burden due to changes in a risk factor (e.g. the reduction in smoking after anti-tobacco interventions), but these tools need estimates of the COPD incidence and prevalence within a given population, among other information, as inputs.

In the present study, we evaluated incidence trends of pre-bronchodilator airflow obstruction (AO) by sex and lifetime smoking history, among adults without asthma. To fulfil this purpose, we analysed 20-year follow-up data from the European Community Respiratory Health Survey (ECRHS)^8–10^ and the Swiss Cohort Study on Air Pollution and Lung and Heart Diseases in Adults (SAPALDIA)^11–13^, and we identified AO using internally-derived equations of the lower limit of normal [LLN; 5^th^ percentile of the distribution of the forced expiratory volume in 1 second (FEV_1_) and the forced vital capacity (FVC) ratio in normal subjects]. In addition, we quantified the extent of misclassification in the definition based on pre-bronchodilator spirometry (using post-bronchodilator measurements from a subsample of our subjects) and we used this information to estimate the overall incidence of post-bronchodilator airflow obstruction (AO_post-BD_), which is the primary characteristic of COPD.

## Results

### Main characteristics of the subjects

**Figure 1:**
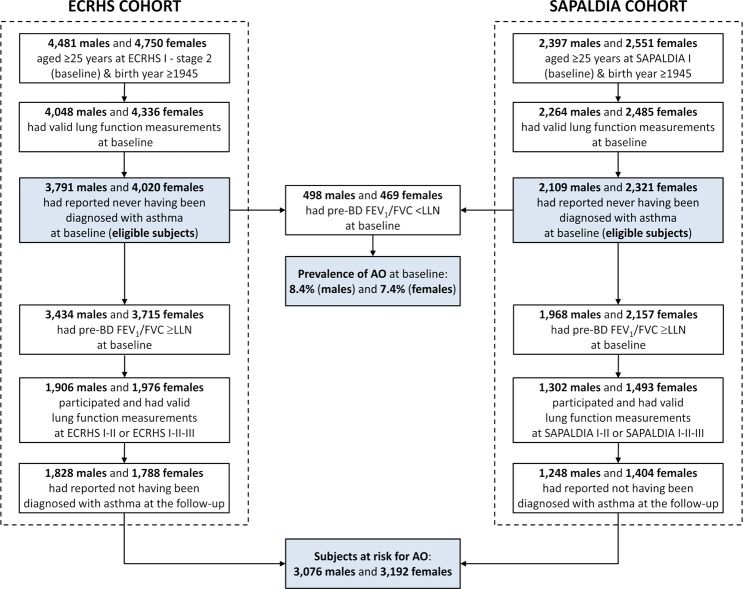
Selection of the subjects at risk for AO. Pre-BD: pre-bronchodilator.

At baseline, 5,900 men and 6,341 women without diagnosed asthma from 32 centres located in 11 European countries (listed in Supplementary Table S1) participated in the ECRHS and SAPALDIA studies, and were eligible for the present study (Fig. 1). Among these individuals, the prevalence of AO was 8.4% in males and 7.4% in females. We identified 3,076 men and 3,192 women (mean age at baseline: 36 years), who had reported not having been diagnosed with asthma at the follow-up, as subjects at risk for AO and we included them in the analyses (Fig. 1 and Table 1). About 60% of these subjects participated and provided valid lung function measurements at all three examinations. The mean duration of the follow-up was 15 years (range: 1–20 years) for both sexes. Among those at risk for AO, 65.9% of men and 56.5% of women were ever smokers at baseline and/or during the follow-up. The median number of pack-years among male and female ever smokers was 20 and 12, respectively. The selection of the subjects at risk for AO and their main characteristics are reported separately for the ECRHS and SAPALDIA studies in Supplementary Tables S2 and S3. The main characteristics of the eligible subjects included or excluded from the analyses are described separately for the two cohorts in Supplementary Tables S4 and S5.

**Table 1:** Main characteristics of the subjects at risk for AO.

N° of subjects	Males	Females
	3,076	3,192
Age at baseline (years), mean (range)	36 (25–48)	36 (25–47)
Subjects who participated and had valid lung function measurements at all examinations, %	59.5	60.6
Duration of the follow-up (years)*, mean (range)	15 (1–20)	15 (1–20)
Ever smokers at baseline and/or follow-up, %	65.9	56.5
N° of lifetime pack-years among ever smokers, median (IQR)	20 (9–34)	12 (4–23)
**Pre-bronchodilator FEV**_**1**_**/FVC(%), mean** ± **s.d**.		
1^st^ examination (baseline)	79.8 ± 5.1	83.2 ± 5.2
2^nd^ examination	77.3 ± 5.6	78.8 ± 5.7
3^rd^ examination	75.2 ± 5.6	76.2 ± 5.2

IQR: interquartile range; s.d.: standard deviation. *The end of the follow-up was the estimated calendar year of AO onset for the incident cases and the calendar year of the last examination for the remaining subjects.

### Incidence of AO

During the follow-up, 208 incident cases of AO out of a total of 45,536 person-years were identified among males, whereas the new cases were 194 out of a total of 48,135 person-years among females (Table 2). The overall incidence rate (IR) of AO in subjects aged 25–64 was 4.4 cases/1,000/year [95% confidence interval (95% CI: 3.5–5.3)] for males and 3.8 (3.1–4.6) for females. The incidence of AO was higher for ever smokers compared to never smokers in both men [incidence rate ratio (IRR) for smoking (95% CI): 2.34 (1.65–3.32)] and women [1.78 (1.31–2.42)]. These estimates are reported separately for the ECRHS and SAPALDIA studies in Supplementary Table S6.

**Table 2:** Incidence of AO.

	Males	Females
**Overall incidence rate**		
N° of incident cases	208	194
person-years at risk	45,536	48,135
cases/1,000/year (95% CI)	4.4 (3.5–5.3)	3.8 (3.1–4.6)
**Incidence rate among never smokers**		
N° of incident cases	39	61
person-years at risk	16,094	21,451
cases/1,000/year (95% CI)	2.3 (1.5–3.2)	2.7 (1.9–3.4)
**Incidence rate among ever smokers**		
N° of incident cases	168	133
person-years at risk	29,368	26,657
cases/1,000/year (95% CI)	5.5 (4.3–6.6)	4.8 (3.8–5.8)

### Trends in the incidence of AO

**Figure 2:**
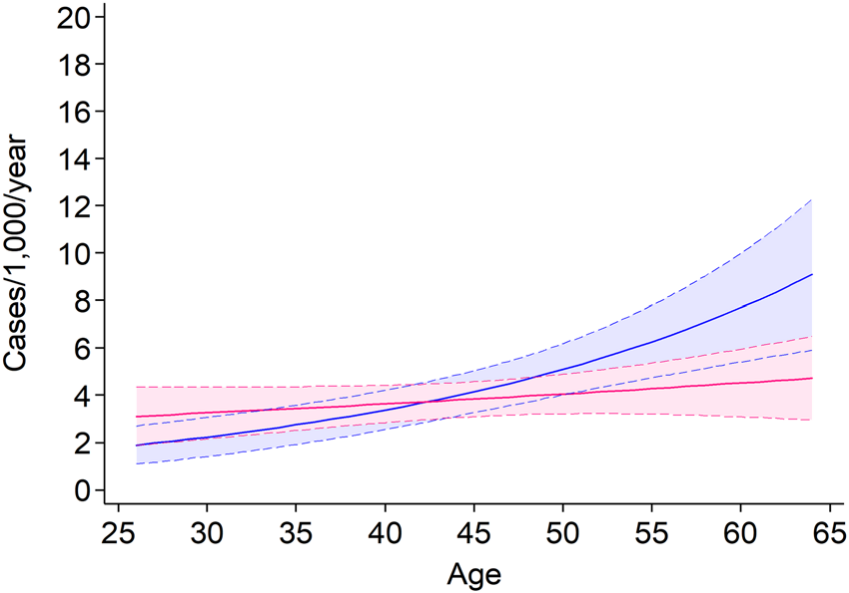
Age trends in AO incidence among males (blue line) and females (red line). Dotted lines represent the 95% confidence limits.

**Figure 3:**
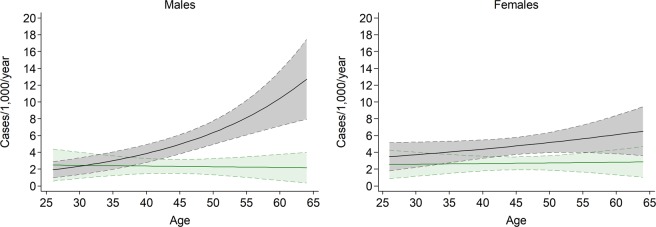
Age trends in AO incidence among ever smokers (black line) and never smokers (green line), according to sex. Dotted lines represent the 95% confidence limits.

**Figure 4:**
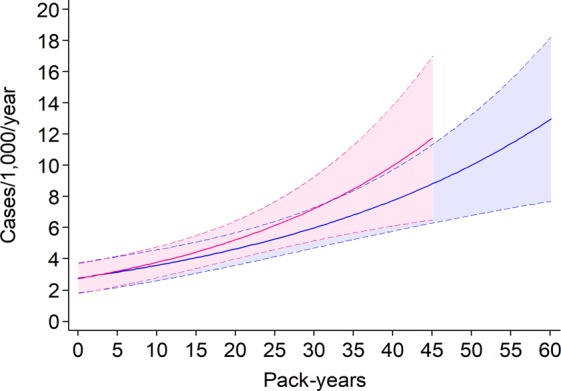
Relationship between AO incidence and lifetime pack-years among male ever smokers (blue line) and female ever smokers (red line). Dotted lines represent the 95% confidence limits.

The incidence of AO greatly increased with ageing in men [IRR per 1-year increase (95% CI): 1.04 (1.02–1.06)], whereas a less steep, and statistically non-significant increase was observed in women [1.01 (0.99–1.03)] (Fig. 2). Among ever smokers, a positive trend in AO incidence was found in men as age increased [1.05 (1.03–1.07)], whereas this age trend was lees steep and did not reach statistical significance in women [1.02 (0.99–1.04)] (Fig. 3). In addition, a strong exposure-response relationship was found between lifetime pack-years and AO incidence for both males [IRR per 1-pack-year increase (95% CI): 1.03 (1.02–1.04)] and females [1.03 (1.02–1.05)] (Fig. 4). Among never smokers, no age trend in AO incidence was observed for either men [IRR per 1-year increase (95% CI): 1.00 (0.96–1.03)] or women [1.00 (0.97–1.03)] (Fig. 3).

### Incidence of AO_post-BD_

The positive predictive value of AO for AO_post-BD_ was 59.1% (95% CI: 52.0–66.2%) among the 212 men and 42.6% (35.1–50.1%) among the 184 women with AO and valid post-bronchodilator spirometry at the 3^rd^ examination. Based on these figures, the estimated IR of AO_post-BD_ was 2.6 (95% CI: 1.7–3.4) cases/1,000/year in males and 1.6 (1.0–2.2) cases/1,000/year in females.

## Discussion

This 20-year follow-up study of two large, population-based, European cohorts of young adults without asthma provided estimates of the incidence of AO over time. This information was used to evaluate the incidence of AO_post-BD_. We found a considerable incidence of AO, quantified as 4.4 male and 3.8 female cases/1,000/year. AO incidence increased with ageing and these trends seem to be largely attributable to active smoking. The exposure-response relationship between lifetime pack-years and AO incidence could possibly be stronger in females. The incidence of AO was also high (2.3 male and 2.7 female cases/1,000/year) among lifetime non-smokers. The expected incidence of AO_post-BD_ was 2.6 and 1.6 cases/1,000/year for males and females, respectively.

Few spirometry-based studies have investigated the incidence of AO in large cohorts with long follow-up periods in Europe^14,15^. Our estimates of the overall incidence of AO are consistent with earlier results from the ECRHS I and II data^14^, in which the incidence of pre-bronchodilator FEV_1_/FVC <70% over 9 years was 3.2 and 2.4 cases/1,000/year in men and women aged 20–54, respectively. The present analyses add further and stronger evidence that AO is a major health problem in adults^16^, especially in males^15^. In respect to the previous analysis of the ECRHS data^14^, the longer follow-up and the inclusion of the SAPALDIA cohort in the present study (with the consequent higher sample size) enabled us to investigate incidence trends across all ages of adulthood. As a result, we found an increase of AO incidence up to 64 years in both sexes, even if this increase was less steep and did not reach statistical significance in women.

The higher incidence of AO with ageing is well known, even if it is unclear whether ageing-related biological mechanisms lead to COPD or if age reflects the accumulation of exposures during a lifetime^17^. In our study, we found that the incidence of AO increased with ageing among ever smokers only, which supports the concept that age reflects cumulative exposures (especially tobacco smoking) throughout life. Therefore, in the present analyses, the higher incidence of AO in men could be due to both the higher percentage of ever smokers and the higher level of tobacco exposure (pack-years) in the male cohort.

We found that female smokers reached the same level of AO incidence at a lower number of pack-years than male smokers, even if our study included a limited number of heavy smokers. Other studies have suggested that women may be at a greater risk of smoking-induced lung function impairment for the same level of exposure as men^18^. This may be related to a sex-specific predisposition for smoking-related lung damage or a dose-dependent effect due to smaller airways in women, with each cigarette smoked representing a proportionally greater exposure. It has been hypothesised that oestrogens could potentiate the oxidative stress due to smoking exposure, contributing to airway remodelling^19,20^. Sørheim and colleagues have also found that a greater lung function reduction and a more severe COPD was more common among female patients with a low smoking exposure^21^.

Current trends in smoking habits may have a complex effect on future trends in AO incidence. During the past decades, smoking initiation rates reduced or levelled off among late adolescents and young adults^22^, and smoking cessation rates increased^23^ across Europe. However, after 1990, smoking initiation rates markedly increased among early adolescents in different European regions^22^. In addition, subjects who start smoking before the age of 16 may be less likely to quit than those who start later^23^. Accordingly, we can speculate an increase in AO incidence during the next decades across Europe among the individuals who were in their early adolescence after the Nineties.

The high incidence of AO among never smokers, which was relatively constant with ageing, supports previous evidence that COPD also generates a substantial burden in the population not exposed to active smoking^24,25^. Early life insults, such as tobacco exposure *in utero* and childhood, low birth weight and childhood lung infections, may increase the risk of COPD^26^. Occupational exposures to chemicals, dust or fumes^27^, indoor exposure to biomass fuels^28^, outdoor air pollution^29^, chronic asthma^30^ and alpha-1-antitrypsin deficiency^31^ may also increase the risk of COPD in subjects who have never smoked.

The positive predictive value of AO for AO_post-BD_ was lower in women than in men in our sample, which could be explained by differences among the subjects with AO. As reported by Sawalha and colleagues^5^, we also found that the percentage of ever smokers (79.7% *vs* 73.4%), the median number of lifetime pack-years (25 *vs* 20), and the percentage of subjects with productive cough (21.0% *vs* 14.5%), factors that may predict the occurrence of COPD^14^, were higher in men than in women with AO and valid post-bronchodilator spirometry at the 3^rd^ examination. In addition, our estimates of the positive predictive value (59.1% in males and 42.6% in females) were lower than the figure obtained by Schermer and colleagues (74.7% in both sexes) when the fixed cut-off definition of AO was used^4^. The fixed cut-off criterion leads to a substantial over-diagnosis of AO in middle-aged and elderly subjects^4^ and, consequently, to a higher positive predictive value.

We found that the expected overall incidence of AO_post-BD_ was half of the incidence of AO. We acknowledge that the expected incidence of AO_post-BD_ could still be an overestimate of the incidence of COPD in Europe, as AO_post-BD_ cases may include a non-negligible proportion of asymptomatic never smokers^32^ who would likely not be diagnosed with COPD in a clinical setting^2^.

The present study has several strengths. Firstly, it relies on the long follow-up of two large population-based cohorts of adults without asthma. In addition, the young age of our subjects at baseline enabled us to investigate AO in the early phases of COPD, which is crucial in identifying the group of individuals who could benefit from preventive interventions. The exclusion of the individuals who had reported a diagnosis of asthma at baseline or at the follow-up should have limited the bias due to the asthma-COPD misclassification. Asthma and COPD have a different aetiology, characteristics and clinical course^33^, and AO_post-BD_ in adulthood (even in smokers) should not be considered as COPD without giving consideration to earlier asthma^34^. However, among the incident cases of AO at the 2^nd^ examination, 61.3% of males and 66.7% of females had either AO or key indicators of COPD^2^ or they reported a physician diagnosis of COPD at the 3^rd^ examination (detailed description available in the Supplementary Information, page 7). This result suggests that a subgroup of AO cases in the present study could be patients with undiagnosed asthma. Further, the inclusion in the analyses of two large cohorts of subjects from 11 countries should have increased the generalizability of our results to the European population. Lastly, the computation of the LLN equations within our cohorts, by taking the heterogeneity of the spirometric measurements among centres into account^35^, should have reduced the misclassification due to the use of predictive equations generated from other populations^36^.

The main limitation of the present study is the lack of post-bronchodilator spirometry at all examinations, which did not enable us to directly estimate the incidence of AO_post-BD_ and to confirm the presence of AO_post-BD_ in subsequent spirometry tests^37,38^. In addition, having measured lung function only at three time points over 20 years could have affected the estimates of the incidence trends, as we could only predict the year of AO onset between two examinations. Further, only 60% of the subjects in our cohorts participated and had valid lung function measurements at all examinations, and attrition could have influenced our findings and limited their generalizability to some extent. However, in males, the overall IR of AO estimated from the subsample of subjects with data from all the examinations was comparable with the figure obtained from the whole cohort (4.5 *vs* 4.4 cases/1,000/year), whereas a lower value was found in females (3.0 *vs* 3.8 cases/1,000/year). The percentage of ever smokers was lower among the eligible subjects included in the study than among those excluded, for both cohorts and sexes (Supplementary Tables S4 and S5). Accordingly, we speculate that AO incidence could be underestimated in our analyses as a consequence of the lower percentage of ever smokers among the study subjects. Lastly, we acknowledge that ever smoking should be subdivided into current or past exposures, and that these two categories should be further subdivided based on pack-years (and time since smoking cessation for quitters), in order to better assess the impact of current and past smoking on AO incidence trends. Unfortunately, these analyses would require a high number of incident cases to obtain stable results.

In conclusion, the incidence of AO was considerable over 20 years among European adults without asthma, who were followed up between ages 25 and 64. The ageing-related increase in AO incidence among ever smokers was strongly related to cumulative tobacco exposure, which seems to largely explain the different age trends for men and women. The exposure-response relationship between lifetime pack-years and AO incidence could possibly be stronger in females. Although the incidence of AO was relatively constant with age, it was high even among lifetime non-smokers, which supports the fact that COPD also represents a substantial burden in the population not exposed to active smoking. The incidence of AO_post-BD_ is expected to be half of the incidence of AO and the extent of misclassification (when using pre-bronchodilator as opposed to post-bronchodilator spirometry) was higher in women.

## Methods

### Study design

The ECRHS and SAPALDIA studies share a comparable research protocol with information collected at three examinations. ECRHS (www.ecrhs.org) is an international, population-based, cohort study on respiratory health on random samples of subjects aged 20–44 years in 1991–1993 (ECRHS I; 1^st^ examination)^8^. Each participant was sent a brief screening questionnaire (stage 1) and, from those who responded, a 20% random sample was invited to undergo a more detailed clinical examination (stage 2). The participants in the ECRHS I - stage 2 were followed up in 1998–2002 (ECRHS II; 2^nd^ examination)^9^ and in 2010–2013 (ECRHS III; 3^rd^ examination)^10^. SAPALDIA (www.sapaldia.ch/en/) is a Swiss, population-based, cohort study on the long-term health effects of air pollutants in subjects aged 18–60 years in 1991, who were randomly selected from local registries of inhabitants to undergo standardized clinical tests (SAPALDIA I; 1^st^ examination)^11,39^. The participants in SAPALDIA I were re-examined in the clinical centres in 2001–2003 (SAPALDIA II; 2^nd^ examination)^12^ and in 2010–2011 (SAPALDIA III; 3^rd^ examination)^13^.

The subjects in both the ECRHS and SAPALDIA studies underwent a detailed clinical interview and pre-bronchodilator spirometry at each examination, and post-bronchodilator spirometry was also measured at the last follow-up contact. The maximum FEV_1_ and the maximum FVC from at least two technically satisfactory manoeuvres were measured according to the American Thoracic Society criteria for repeatability^39,40^. Biomedin, Jaeger Masterscope, SensorMedics or Vitalograph spirometers were used at the 1^st^ and 2^nd^ examinations, whereas NDD EasyOne was used in almost all centres at the 3^rd^ examination (see Supplementary Table S1). In both the studies, the lung function measurements were corrected for the change in spirometer, according to Bridevaux and colleagues^41^.

### Selection of the subjects and definitions

The *eligible subjects* (Fig. 1) were those who:

- were at least 25 years old in the ECRHS - stage 2 or SAPALDIA I, in order to exclude those who may still not have reached the plateau phase of lung function growth at baseline^42^;
- were born in 1945 or later, in order to have the same age range in the ECRHS and SAPALDIA cohorts;
- had provided valid lung function measurements at baseline;
- had reported never having been diagnosed with asthma at baseline.

The *subjects at risk for AO* (Fig. 1) were the eligible participants who:

- had pre-bronchodilator FEV_1_/FVC ≥LLN at baseline (internally-derived LLN equations were computed, which take the variation in the spirometric measurements due to differences among centres into account^35,43^; detailed description available in the Supplementary Information, pages 3–4);
- had provided valid lung function measurements during at least two consecutive examinations (ECRHS/SAPALDIA I-II or ECRHS/SAPALDIA I-II-III);
- had reported not having been diagnosed with asthma at the follow-up.

The subjects at risk who had pre-bronchodilator FEV_1_/FVC <LLN at one of the follow-up contacts were considered to be *incident cases of AO*. For each new case, the year of age at AO onset was estimated by linear interpolation, (i) assuming that the decline in FEV_1_/FVC between two examinations was constant and (ii) taking into account the change in the LLN cut-off with ageing (detailed description available in the Supplementary Information, page 5). Based on these assumptions, we obtained the year of age when the FEV_1_/FVC had reached a level below the LLN for each individual. The end of the follow-up was the calendar year of AO onset (obtained by summing the estimated year of age at onset to the birth year) for the incident cases, and the calendar year of the last examination for the remaining subjects.

Smoking was classified as “*never”* (i.e. never smoking at baseline and follow-up) and “*ever”* (i.e. past or current smoking at baseline or follow-up). Lifetime pack-years were quantified at the end of the follow-up.

### Statistical analyses

The ECRHS and SAPALDIA data were pooled to estimate the IRs of AO and their trends, within age 25–64 years during 1991–2011. Data pooling was justified due to the fact that the AO rates were similar in the two studies for both sexes (see Supplementary Table S6). Age was right-censored at 64 years in order to avoid data sparseness, whereas the time period was right-censored at 2011 in order to analyse the data from the same period for both the studies. The sex-specific pooled datasets were reshaped by age to estimate AO incidence (detailed description available in the Supplementary Information, page 6).

#### Incidence of AO

The overall IRs of AO were computed separately in males and females using two-level Poisson models (subject = level 1 unit; centre = level 2 unit). All the models had log person-years as the offset, a random intercept term at level 2 and study (0 = ECRHS, 1 = SAPALDIA) as a fixed effect. The smoking-specific IRs of AO were estimated adding smoke (ever *vs* never) and the smoke × study interaction term to the fixed part of the models. All the rates were obtained by setting the indicator of the study equal to the proportion of person-years in SAPALDIA (0.44 for males and 0.46 for females), in order to account for the different overall time at risk in the two cohorts.

#### Trends in the incidence of AO

Age trends in AO incidence were estimated separately in males and females using two-level Poisson models with log person-years as the offset, a random intercept term at level 2 and age, study and the age × study interaction term as fixed effects. Age was included as a linear factor in the models since higher degree polynomials or spline interpolations did not improve goodness-of-fit. Age trends were computed according to smoking history adding smoke and the age × smoke interaction term to the fixed part of the models. Lastly, AO incidence was evaluated according to lifetime pack-years among ever smokers, using sex-specific reshaped datasets by pack-years and two-level Poisson models with pack-years, study and the pack-years × study interaction term as fixed effects. The number of pack-years was right-censored at 60 for men and 45 for women to avoid data sparseness.

#### Incidence of AO_post-BD_

The expected IR of AO_post-BD_ (post-bronchodilator FEV_1_/FVC <LLN) was estimated from the overall IR of AO, separately in men and women. Firstly, the positive predictive value of AO for AO_post-BD_ was computed for each sex among those with pre-bronchodilator FEV_1_/FVC ≥LLN and valid post-bronchodilator lung function measurements in the ECRHS/SAPALDIA III. Secondly, the expected IR of AO_post-BD_ was obtained as the product of the sex-specific estimates of the AO rate and the positive predictive value. The 95% confidence interval of the AO_post-BD_ rate was computed by using the delta method and assuming that the sex-specific estimates of the AO rate and of the positive predictive value have a perfect positive correlation.

The statistical analyses were carried out using STATA 15 (StataCorp, College Station, TX).

### Ethics statement

Ethics approval was obtained by all centres from the appropriate ethics committees in ECRHS [Antwerp City and Antwerp South: Adviescommissie Medische Ethiek UZA-UA (CME); Tartu: Research Ethics Committee of the University of Tartu, Estland (N° 209T-17); French centres: Comite de protection des personnes, Sud V Est (N° 2011-A00013-38); German centres: Ethik-Kommission der Bayerischen Landesarztekammer (N° 10015); Reykjavik: National Biotecs Committe of Iceland (NBCI) (N° VSNb2011090016/03.11); Pavia: Fondazione IRCCS Policlinico ‘San Matteo’ (N° P-20110024215); Turin: Comitato Etico dell’Azienda Sanitaria Locale TO/2 di Torino (N° 569/09/08); Verona: Comitato Etico per la Sperimentazione dell’Azienda Ospedaliera Istituti Ospitalieri di Verona (N° 1393); Bergen: Universitetet i Bergen, Regional komité for medisinsk og helsefaglig forskningsetikk, Vest-Norge (REK Vest) (N° 2010/759); Albacete: Comité de Ética e Investigación de Complejo Hospitalario de Albacete (N° 04/09); Barcelona: Comité Ético de Investigación Clínica del Instituto Municipal de Asistencia Sanitaria, Barcelona, Spain (N° PS09/00716); Galdakao: Comité Ético de Investigación del Hospital de Galdakao, Spain (N° 20101104); Huelva: Comisión de Investigación del Hospital Juan Ramón Jiménez de Huelva (N° 20090417); Oviedo: Comité Ético de Investigación Clínica Regional, Hospital Universitario Central de Asturias (N° 20110415); Swedish centres: Ethics Committee at the Medical Faculty, Uppsala University (N° 1999/313 and 2010/068); Basel: Swiss Academy of Medical Sciences and the ethics committee of Basel (N° PV123/00,157/00); UK centres: NRES Committee London - Stanmore (REC Reference 11/LO/0965 IRAS number 70769)] and in SAPALDIA [Swiss Academy of Medical Sciences and the regional committees for each study centre; ethics approval is coordinated by the lead ethical agency EKNZ in Basel, Switzerland]. In both the studies, written informed consent was obtained from the participants and all experiments were performed in accordance with relevant guidelines and regulations.

## Supplementary information

Supplementary Information

## Data Availability

Due to data protection reasons, the datasets analysed in the present study cannot be made publicly available. The datasets are available to interested researchers from the corresponding author on reasonable request.
