# Supplementary material for: Incidence trends of airflow obstruction among European adults without asthma: a 20-year cohort study
Source: Sci Rep. 2020 Feb 26;10:3452. doi: 10.1038/s41598-020-60478-5 (PMC7044325; doi:10.1038/s41598-020-60478-5)
Supplement: Supplementary file 1 — Supplementary Information [file 41598_2020_60478_MOESM1_ESM.pdf]

SUPPLEMENTARY INFORMATION

Incidence trends of airflow obstruction among European adults without asthma: a 20-year cohort study

Simone Accordini[1,*], Lucia Calciano[1], Alessandro Marcon[1], Giancarlo Pesce[1,2], Josep M Antó[3,4,5,6], Anna B Beckmeyer-Borowko[7,8], Anne-Elie Carsin[3,5,6], Angelo G Corsico[9], Medea Imboden[7,8], Christer Janson[10], Dirk Keidel[7,8], Francesca Locatelli[1], Cecilie Svanes[11,12], Peter GJ Burney[13,14], Deborah Jarvis[13,14,¶], Nicole M Probst-Hensch[7,8,¶], Cosetta Minelli[13,¶].

*Corresponding author; [¶]equal senior authors.

[1]Unit of Epidemiology and Medical Statistics, Department of Diagnostics and Public Health, University of Verona, Verona, Italy; [2]Sorbonne Universités, INSERM UMR-S 1136, IPLESP, Team EPAR, F75012, Paris, France; [3]ISGlobal, Centre for Research in Environmental Epidemiology (CREAL), Barcelona, Spain; [4]Hospital del Mar Medical Research Institute (IMIM), Barcelona, Spain; [5]CIBER Epidemiología y Salud Pública (CIBERESP), Barcelona, Spain; [6]Universitat Pompeu Fabra (UPF), Barcelona, Spain; [7]Department of Epidemiology and Public Health, Swiss Tropical and Public Health Institute, Basel, Switzerland; [8]University of Basel, Basel, Switzerland; [9]Division of Respiratory Diseases, IRCCS 'San Matteo' Hospital Foundation-University of Pavia, Pavia, Italy; [10]Department of Medical Sciences: Respiratory, Allergy and Sleep Research, Uppsala University, Uppsala, Sweden; [11]Centre for International Health, Department of Global Public Health and Primary Care, University of Bergen, Bergen, Norway; [12]Department of Occupational Medicine, Haukeland University Hospital, Bergen, Norway; [13]Population Health and

Occupational Disease, National Heart and Lung Institute, Imperial College London, London, UK; [14]MRC-PHE Centre for Environment and Health, Imperial College London, London, UK.

CORRESPONDING AUTHOR:

Professor Simone Accordini, Sezione di Epidemiologia e Statistica Medica, Dipartimento di Diagnostica e Sanità Pubblica, Università degli Studi di Verona c/o Istituti Biologici II, Strada Le Grazie 8, 37134 Verona, Italy; phone: +39 045 8027657; e-mail: simone.accordini@univr.it; ORCID iD: 0000-0003-1510-6193

Internally-derived LLN equations

At each examination, pre-bronchodilator FEV_1/FVC values (corrected for the change in spirometer according to Bridevaux and colleagues [reference 41 in the manuscript]) were included in the LLN computations if obtained from normal putative subjects aged 25-64, who had provided valid lung function measurements during at least two consecutive examinations. Normal individuals were those who had reported none of the following conditions at the time of the measurement:

- chronic cough or phlegm;
- asthma-like symptoms [wheezing, nocturnal shortness of breath, shortness of breath at rest, shortness of breath following strenuous activities, nocturnal chest tightness] in the previous 12 months;
- use of medicines for breathing problems (in the ECRHS I-II-III and SAPALDIA I) or use of medicines for asthma (in the SAPALDIA II-III) in the previous 12 months;
- asthma (with or without a physician diagnosis) during a lifetime;
- smoking during lifetime.

Accordingly, 1,218 and 1,496 FEV_1/FVC values from 635 and 786 normal men and women in ECRHS, and 1,038 and 1,238 FEV_1/FVC values from 481 and 621 normal men and women in SAPALDIA were selected, respectively.

Three-level linear regression models (measurement: level 1 unit; subject: level 2 unit; centre: level 3 unit) were used to calculate the LLN equations separately for males and females in the ECRHS and SAPALDIA studies. The models had random intercept terms at levels 2 and 3, a random slope for age at level 2, an unstructured variance-covariance matrix of the random effects at level 2, a 1st order autoregressive error at level 1, and examination (1st, 2nd or 3rd measurement), the age × examination interaction term and height as fixed effects. Age was included as

a fixed effect in the model for males in ECRHS because the variance of the random slope was negligible. The LLN equations were computed as follows:

$$\text{LLN(examination, age, height)} = \mathit{FEV_1/FVC}^{\text{hat}} - \mathit{z\text{-}score}_{0.05} \cdot \sqrt{[\sigma_{(1)}^2 + \sigma_{(2)}^2 + \sigma_{(3)}^2]}$$

where:

- $\mathit{FEV_1/FVC}^{\text{hat}} = b_0 + b_1 \cdot \mathit{examination} + b_2 \cdot \mathit{age} + b_3 \cdot \mathit{age} \cdot \mathit{examination} + b_4 \cdot \mathit{height}$ is the predicted FEV_1/FVC obtained from the fixed part intercept (b_0, b_1) and slope coefficients (b_2-b_4; *age* is the age of a subject at a given examination and *height* is the height of a subject at the 2nd examination);
- $\mathit{z\text{-}score}_{0.05}$ is the 5th percentile of the z-score of FEV_1/FVC;
- $\sigma_{(1)}^2 = \sigma_{residual}^2 / (1 - \rho^2)$ is the variance at level 1 ($\sigma_{residual}^2$ is the residual variance and ρ is the 1st order autocorrelation coefficient);
- $\sigma_{(2)}^2 = \sigma_{intercept}^2 + \sigma_{slope}^2 \cdot \mathit{age}^2 + 2 \cdot \sigma_{intercept,\ slope} \cdot \mathit{age}$ is the variance at level 2 ($\sigma_{intercept}^2$, σ_{slope}^2 and $\sigma_{intercept,\ slope}$ are the components of the variance-covariance matrix of the random effects at level 2; $\sigma_{slope}^2 = \sigma_{intercept,\ slope} = 0$ for males in ECRHS);
- $\sigma_{(3)}^2$ is the variance at level 3.

The estimates of the fixed and random parameters were obtained using the restricted maximum likelihood (REML) method.

Year of age at AO onset

For each new case, the year of age at AO onset (t_{AO}) was estimated by linear interpolation as follows:

- FEV_1/FVC was obtained for each year of age (t) between the year of age at the last examination with normal spirometry (t_1) and the year of age at the subsequent examination with AO (t_2) assuming a linear decrease:

  $$FEV_1/FVC(t) = FEV_1/FVC(t_1) + [FEV_1/FVC(t_2) – FEV_1/FVC(t_1)] \cdot [(t – t_1) / (t_2 – t_1)];$$

- LLN was obtained for each year of age (t) between t_1 and t_2 assuming a linear decrease:

  $$LLN(t) = LLN(t_1) + [LLN(t_2) – LLN(t_1)] \cdot [(t – t_1) / (t_2 – t_1)];$$

- t_{AO} was the first year of age between t_1 and t_2 with $FEV_1/FVC(t_{AO}) < LLN(t_{AO})$.

As a result of the interpolation process, the incident cases were aged 26 or older.

Reshaped datasets

When computing the IRs of AO, we used a reshaped dataset by age ("long" format) for each sex. The "original" dataset, which contains one record for each subject, was reshaped by replicating each individual record in as many records as the number of age years from the subject's first participation in ECRHS or SAPALDIA (or from age 25 if his/her age at the first study was <25) to the end of follow-up (or to age 64 if his/her age at the end of follow-up was >64). The end of follow-up was the calendar year of AO onset for the incident cases, and the calendar year of the last examination for the remaining subjects. The "new" record of a subject at a given age contained the subject's age and health indicator at that age (i.e. the dummy variable indicating if he/she was an incident case at that age). Therefore, a subject contributed one person-year to the denominator of the IR for each age year without AO, and one unit to the numerator and one person-year to the denominator of the IR for the age year when he/she developed AO. When estimating the trends in AO incidence as a function of lifetime pack-years among ever smokers, reshaping was carried out by pack-years instead.

Stability of AO

We evaluated how many incident cases of AO at ECRHS/SAPALDIA II had one of the following conditions at ECRHS/SAPALDIA III:

- pre-bronchodilator FEV_1/FVC <LLN;
- pre-bronchodilator FEV_1/FVC ≥LLN AND ever smoking (≥10 pack-years) AND key respiratory symptoms for COPD (chronic cough, chronic sputum production, dyspnoea, shortness of breath following strenuous activity in the last 12 months);
- pre-bronchodilator FEV_1/FVC ≥LLN AND self-reported diagnosis of chronic bronchitis, emphysema or COPD;
- pre-bronchodilator FEV_1/FVC ≥LLN AND father and/or mother with a diagnosis of chronic bronchitis, emphysema or COPD.

Among the incident cases of AO at the 2nd examination, the subjects who had fulfilled at least one of these conditions at the 3rd examination were: 57/93 (61.3%) males and 44/66 (66.7%) females.

Table S1. Brand of the spirometers* used by study, centre and examination.

Study	Country	Centre	1st examination	2nd examination	3rd examination
ECRHS	Belgium	Antwerp City	SensorMedics (D)	Jaeger Masterscope	NDD EasyOne
		Antwerp South	SensorMedics (D)	Jaeger Masterscope	NDD EasyOne
	Estonia	Tartu	Jaeger Masterscope	Jaeger Masterscope	NDD EasyOne
	France	Bordeaux	Vitalograph	Vitalograph	NDD EasyOne
		Grenoble	Biomedin	Biomedin	NDD EasyOne
		Montpellier	Biomedin	Biomedin	NDD EasyOne
		Paris	Biomedin	Biomedin	NDD EasyOne
	Germany	Erfurt	Jaeger Masterscope	Jaeger Masterscope	NDD EasyOne
		Hamburg	Jaeger Masterscope	Jaeger Masterscope	NDD EasyOne
	Iceland	Reykjavik	SensorMedics (D)	SensorMedics (D)	NDD EasyOne
	Italy	Pavia	Biomedin	Biomedin	NDD EasyOne
		Turin	Biomedin	Biomedin	Biomedin
		Verona	Biomedin	Biomedin	Biomedin
	Norway	Bergen	SensorMedics (D)	SensorMedics (D)	NDD EasyOne
	Spain	Albacete	Biomedin	Biomedin	NDD EasyOne
		Barcelona	Biomedin	Biomedin	NDD EasyOne
		Galdakao	Biomedin	Biomedin	NDD EasyOne
		Huelva	Biomedin	Biomedin	NDD EasyOne
		Oviedo	Biomedin	Biomedin	NDD EasyOne
	Sweden	Gothenburg	SensorMedics (D)	SensorMedics (D)	NDD EasyOne
		Umea	SensorMedics (D)	SensorMedics (D)	NDD EasyOne
		Uppsala	SensorMedics (D)	SensorMedics (D)	NDD EasyOne
	United Kingdom	Ipswich	Biomedin	Biomedin	NDD EasyOne
		Norwich	Biomedin	Biomedin	NDD EasyOne
SAPALDIA	Switzerland	Aarau	SensorMedics (H)	SensorMedics (H)	NDD EasyOne
		Basel†	SensorMedics (H)	SensorMedics (H)	NDD EasyOne
		Davos	SensorMedics (H)	SensorMedics (H)	NDD EasyOne
		Geneva	SensorMedics (H)	SensorMedics (H)	NDD EasyOne
		Lugano	SensorMedics (H)	SensorMedics (H)	NDD EasyOne
		Montana	SensorMedics (H)	SensorMedics (H)	NDD EasyOne
		Payerne	SensorMedics (H)	SensorMedics (H)	NDD EasyOne
		Wald	SensorMedics (H)	SensorMedics (H)	NDD EasyOne

* SensorMedics (D) is a volume-displacement spirometer and SensorMedics (H) is a heated-wire spirometer.

† The subjects from Basel participating in ECRHS, who were also recruited for SAPALDIA, were considered as part of SAPALDIA throughout the present analysis.

Table S2. Selection of the subjects at risk for AO by study and centre.

			Males			Females		
Study	Country	Centre	Participants*	Eligible subjects†	Subjects at risk for AO‡	Participants*	Eligible subjects†	Subjects at risk for AO‡
ECRHS	Belgium	Antwerp City	156	118	58	195	153	68
		Antwerp South	179	157	88	174	142	91
	Estonia	Tartu	73	44	5	117	86	18
	France	Bordeaux	166	115	26	166	124	30
		Grenoble	217	189	109	201	186	106
		Montpellier	197	166	58	209	173	46
		Paris	259	204	91	301	247	106
	Germany	Erfurt	282	234	87	286	261	80
		Hamburg	397	335	80	407	357	87
	Iceland	Reykjavik	228	215	159	240	214	128
	Italy	Pavia	142	119	70	122	99	58
		Turin	95	82	38	94	76	32
		Verona	134	126	66	135	119	57
	Norway	Bergen	343	311	195	338	307	180
	Spain	Albacete	141	135	58	176	166	67
		Barcelona	107	82	27	120	78	36
		Galdakao	175	150	89	156	127	73
		Huelva	104	101	60	103	90	54
		Oviedo	120	99	56	125	104	49
	Sweden	Gothenburg	242	212	91	263	234	97
		Umea	201	178	105	212	189	94
		Uppsala	224	161	88	215	159	79
	United Kingdom	Ipswich	163	144	72	194	168	78
		Norwich	136	114	52	201	161	74
			4,481	3,791	1,828	4,750	4,020	1,788
SAPALDIA	Switzerland	Aarau	326	283	196	327	295	207
		Basel†	376	337	157	371	330	160
		Davos	201	165	106	243	215	131
		Geneva	274	234	106	255	227	100
		Lugano	253	223	140	336	316	199
		Montana	200	170	119	199	186	125
		Payerne	359	316	151	397	359	198
		Wald	408	381	273	423	393	284
			2,397	2,109	1,248	2,551	2,321	1,404
TOTAL			6,878	5,900	3,076	7,301	6,341	3,192

* Subjects aged ≥25 years at the ECRHS - stage 2 or SAPALDIA I (baseline), who were born in 1945 or later.

† Subjects who had valid lung function measurements at baseline and who reported never having been diagnosed with asthma at baseline.

‡ Subjects who had pre-bronchodilator FEV_1/FVC ≥LLN at baseline, who participated and had valid lung function measurements at ECRHS/SAPALDIA I-II or ECRHS/SAPALDIA I-II-III, and who reported not having been diagnosed with asthma at the follow-up.

Table S3. Main characteristics of the subjects at risk for AO by study.

	Males		Females	
	ECRHS	SAPALDIA	ECRHS	SAPALDIA
N° of subjects	1,828	1,248	1,788	1,404
Age at baseline (years), mean (range)	35 (25-48)	36 (25-46)	35 (25-47)	36 (25-46)
Subjects who participated and had valid lung function measurements at all examinations, %	52.3	70.0	56.0	66.3
Duration of the follow-up (years)*, mean (range)	14 (1-20)	16 (1-20)	14 (1-20)	16 (1-20)
Ever smokers at baseline and/or follow-up, %	67.3	63.9	58.2	54.4
N° of lifetime pack-years among ever smokers, median (IQR)	19 (9-32)	22 (9-36)	11 (4-21)	13 (5-26)
Pre-bronchodilator FEV_1/FVC(%), mean ± s.d.				
1st examination (baseline)	79.7 ± 4.8	80.0 ± 5.5	83.7 ± 5.0	82.6 ± 5.4
2nd examination	78.1 ± 5.2	76.1 ± 5.9	79.8 ± 5.4	77.4 ± 5.8
3rd examination	76.6 ± 5.4	73.6 ± 5.4	76.9 ± 5.2	75.5 ± 5.1

IQR: interquartile range; s.d.: standard deviation.

* The end of the follow-up was the estimated calendar year of AO onset for the incident cases and the calendar year of the last examination for the remaining subjects.

Table S4. Main characteristics of the eligible subjects* included or excluded from the analyses (ECRHS study).

	Males		Females	
	Included†	Excluded‡	Included†	Excluded‡
N° of subjects	2,079	1,712	2,147	1,873
Age at baseline (years), mean (range)	35 (25-48)	35 (25-47)	35 (25-47)	35 (25-47)
Ever smokers at baseline and/or follow-up, %	67.6	69.7	58.8	61.1
N° of lifetime pack-years among ever smokers, median (IQR)	19 (9-32)	15 (7-25)	11 (4-21)	10 (4-18)
Pre-bronchodilator FEV_1/FVC(%) at the 1st examination (baseline), mean ± s.d.	78.5 ± 6.0	78.5 ± 6.5	82.5 ± 6.2	83.1 ± 6.2

IQR: interquartile range; s.d.: standard deviation.

* Subjects who had valid lung function measurements and reported never having been diagnosed with asthma at the ECRHS I - stage 2 (baseline).

† Eligible subjects who participated in ECRHS II or ECRHS II-III, and who had valid lung function measurements and reported information on asthma at the follow-up.

‡ Eligible subjects who did not participate in ECRHS II-III, or who did not have valid lung function measurements or did not report information on asthma at the follow-up.

Table S5. Main characteristics of the eligible subjects* included or excluded from the analyses (SAPALDIA study).

	Males		Females	
	Included†	Excluded‡	Included†	Excluded‡
N° of subjects	1,385	724	1,587	734
Age at baseline (years), mean (range)	36 (25-46)	36 (25-46)	36 (25-46)	36 (25-46)
Ever smokers at baseline and/or follow-up, %	64.1	72.6	55.6	64.4
N° of lifetime pack-years among ever smokers, median (IQR)	21 (9-35)	19 (9-32)	13 (5-26)	14 (7-22)
Pre-bronchodilator FEV_1/FVC(%) at the 1st examination (baseline), mean ± s.d.	79.0 ± 6.7	79.3 ± 7.0	81.7 ± 6.4	81.6 ± 6.9

IQR: interquartile range; s.d.: standard deviation.

* Subjects who had valid lung function measurements and reported never having been diagnosed with asthma at SAPALDIA I (baseline).

† Eligible subjects who participated in SAPALDIA II or SAPALDIA II-III, and who had valid lung function measurements and reported information on asthma at the follow-up.

‡ Eligible subjects who did not participate in SAPALDIA II-III, or who did not have valid lung function measurements or did not report information on asthma at the follow-up.

Table S6. Incidence of AO by study.

	Males				Females			
	ECRHS	SAPALDIA	p-value	Pooled cohorts	ECRHS	SAPALDIA	p-value	Pooled cohorts
Overall incidence rate								
N° of incident cases	124	84	-	208	98	96	-	194
person-years at risk	25,358	20,178	-	45,536	25,816	22,319	-	48,135
cases/1,000/year (95%CI)	4.6 (3.5-5.8)	4.1 (2.7-5.5)	0.56	4.4 (3.5-5.3)	3.7 (2.8-4.5)	4.1 (2.8-5.3)	0.60	3.8 (3.1-4.6)
Incidence rate among never smokers								
N° of incident cases	23	16	-	39	28	33	-	61
person-years at risk	8,499	7,595	-	16,094	11,005	10,446	-	21,451
cases/1,000/year (95%CI)	2.5 (1.4-3.7)	2.1 (0.9-3.3)	0.59	2.3 (1.5-3.2)	2.5 (1.5-3.4)	2.9 (1.7-4.1)	0.55	2.7 (1.9-3.4)
Incidence rate among ever smokers								
N° of incident cases	100	68	-	168	70	63	-	133
person-years at risk	16,785	12,583	-	29,368	14,784	11,873	-	26,657
cases/1,000/year (95%CI)	5.6 (4.2-7.1)	5.3 (3.4-7.1)	0.72	5.5 (4.3-6.6)	4.6 (3.3-5.8)	5.0 (3.4-6.7)	0.82	4.8 (3.8-5.8)

APPENDIX

ECRHS STUDY

ECRHS I

Co-ordinating Centre (London): P Burney, S Chinn, C Luczynska†, D Jarvis, E Lai.

Project Management Group: P Burney (Project leader-UK), S Chinn (UK), C Luczynska† (UK), D Jarvis (UK), P Vermeire† (Antwerp), H Kesteloot (Leuven), J Bousquet (Montpellier), D Nowak (Hamburg), J Prichard† (Dublin), R de Marco† (Verona), B Rijcken (Groningen), JM Anto (Barcelona), J Alves (Oporto), G Boman (Uppsala), N Nielsen (Copenhagen), P Paoletti (Pisa).

Financial support: The following grants helped to fund the local studies. **Australia**: Asthma Foundation of Victoria, Allen and Hanbury's, **Belgium**: Belgian Science Policy Office, National Fund for Scientific Research, **Estonia:** Estonian Science Foundation, grant no 1088, **France:** Ministère de la Santé, Glaxo France, Institut Pneumologique d'Aquitaine, Contrat de Plan Etat-Région Languedoc-Rousillon, CNMATS, CNMRT (90MR/10, 91AF/6), Ministre delegué de la santé, RNSP, France; GSF, **Germany**: Bundesminister für Forschung und Technologie, **Italy:** Ministero dell'Università e della Ricerca Scientifica e Tecnologica, CNR, Regione Veneto grant RSF n. 381/05.93, **Norway:** Norwegian Research Council project no. 101422/310, **Spain:** Fondo de Investigación Sanitaria (#91/0016-060-05/E, #92/0319 and #93/0393), Hospital General de Albacete, Hospital General Juan Ramón Jiménez, Dirección Regional de Salud Pública (Consejería de Sanidad del Principado de Asturias), CIRIT (1997 SGR 00079) and Servicio Andaluz de Salud, **Sweden:** The Swedish Medical Research Council, the Swedish Heart Lung Foundation, the Swedish Association against Asthma and Allergy, **Switzerland:** Swiss national Science Foundation grant 4026-28099, **UK:** National Asthma Campaign, British Lung Foundation, Department of Health, South Thames Regional Health Authority.

Coordination: The co-ordination of this work was supported by the European Commission and the authors and participants are grateful to the late C. Baya and M. Hallen for their help during the study and K. Vuylsteek and the members of the COMAC for their support.

ECRHS II

Steering Committee: U. Ackermann-Liebrich (University of Basel, Switzerland); N. Kuenzli (University of Basel, and University of Southern California, Los Angeles, USA); J.M. Antó and J. Sunyer (Institut Municipal d'Investigació Médica (IMIM-IMAS), Universitat Pompeu Fabra (UPF), Spain); P. Burney (project leader), S Chinn, D. Jarvis, J. Knox and C. Luczynska (King's College London, UK); I. Cerveri (University of Pavia, Italy); R. de Marco† (University of Verona, Italy); T. Gislason (Iceland University Hospital, Iceland); J. Heinrich and M. Wjst (GSF–Institute of Epidemiology, Germany); C. Janson (Uppsala University, Sweden); B. Leynaert and F. Neukirch (Institut National de la Santé et de la Recherche Medicale (INSERM), France); J. Schouten (University of Groningen, The Netherlands); C. Svanes (University of Bergen, Norway); P. Vermeire† (University of Antwerp, Belgium).

Principal Investigators and senior scientific teams: **Australia:** (M. Abramson, E.H. Walters, J. Raven); **Belgium: South Antwerp and Antwerp City** (P. Vermeire, J. Weyler, M. van Sprundel, V. Nelen); **Estonia: Tartu** (R. Jõgi, A. Soon); **France: Paris** (F. Neukirch, B. Leynaert, R. Liard, M. Zureik), **Grenoble** (I. Pin, J. Ferran-Quentin), **Bordeaux** (A. Taytard,

C. Raherison), **Montpellier** (J. Bousquet, P.J. Bousquet); **Germany: Erfurt** (J. Heinrich, M. Wjst, C. Frye, I. Meyer); **Iceland: Reykjavik** (T. Gislason, E. Bjornsson, D. Gislason, K.B. Jörundsdóttir*);* **Italy: Turin** (R. Bono, M. Bugiani, P. Piccioni, E. Caria, A. Carosso, E. Migliore, G. Castiglioni), **Verona** (R. de Marco†, G. Verlato, E. Zanolin, S. Accordini, A. Poli, V. Lo Cascio, M. Ferrari, I. Cazzoletti), **Pavia** (A. Marinoni, S. Villani, M. Ponzio, F. Frigerio, M. Comelli, M. Grassi, I. Cerveri, A. Corsico); **Norway: Bergen** (A. Gulsvik, E. Omenaas, C. Svanes, B. Laerum); **Spain: Albacete** (J. Martinez-Moratalla Rovira, E. Almar, M. Arévalo, C. Boix, G. González, J.M. Ignacio García, J. Solera, J. Damián), **Galdakao** (N. Muñozguren, J. Ramos, I. Urrutia, U. Aguirre), **Barcelona** (J.M. Antó, J. Sunyer, M. Kogevinas, J.P. Zock, X. Basagaña, A. Jaen, F. Burgos, C. Acosta), **Huelva** (J. Maldonado, A. Pereira, J.L. Sanchez), **Oviedo** (F. Payo, I. Huerta, A. de la Vega, L. Palenciano, J. Azofra, A. Cañada); **Sweden**: **Göteborg** (K. Toren, L. Lillienberg, A.C. Olin, B. Balder, A. Pfeifer-Nilsson, R. Sundberg), **Umea** (E. Norrman, M. Soderberg, K.A. Franklin, B. Lundback, B. Forsberg, L. Nystrom), **Uppsala** (C. Janson, G. Boman, D. Norback, G. Wieslander, M. Gunnbjornsdottir); **Switzerland: Basel** (N. Küenzli, B. Dibbert, M. Hazenkamp, M. Brutsche, U. Ackermann-Liebrich); **United Kingdom: Ipswich** (D. Jarvis, R. Hall, D. Seaton), **Norwich** (D. Jarvis, B. Harrison).

Financial Support: **Australia:** National Health and Medical Research Council; **Belgium:** Antwerp: Fund for Scientific Research (grant code, G.0402.00), University of Antwerp, Flemish Health Ministry; **Estonia: Tartu** Estonian Science Foundation grant no 4350; **France: (All)** Programme Hospitalier de Recherche Clinique—Direction de la Recherche Clinique (DRC) de Grenoble 2000 number 2610, Ministry of Health, Ministère de l'Emploi et de la Solidarité, Direction Génerale de la Santé, Centre Hospitalier Universitaire (CHU) de Grenoble, **Bordeaux**: Institut Pneumologique d'Aquitaine, **Grenoble:** Comite des Maladies Respiratoires de l'Isere, **Montpellier:** Aventis (France), Direction Regionale des Affaires Sanitaires et Sociales Languedoc-Roussillon**, Paris:** Union Chimique Belge-Pharma (France), Aventis (France), Glaxo France; **Germany: Erfurt** GSF—National Research Centre for Environment and Health, Deutsche Forschungsgemeinschaft (grant code, FR1526/1-1), **Hamburg:** GSF—National Research Centre for Environment and Health, Deutsche Forschungsgemeinschaft (grant code, MA 711/4-1); **Iceland: Reykjavik**, Icelandic Research Council, Icelandic University Hospital Fund; **Italy: Pavia** GlaxoSmithKline Italy, Italian Ministry of University and Scientific and Technological Research (MURST), Local University Funding for Research 1998 and 1999, **Turin**: Azienda Sanitaria Locale 4 Regione Piemonte (Italy), Azienda Ospedaliera Centro Traumatologico Ospedaliero/Centro Traumatologico Ortopedico—Istituto Clinico Ortopedico Regina Maria Adelaide Regione Piemonte, **Verona:** Ministero dell'Università e della Ricerca Scientifica (MURST), Glaxo Wellcome spa; **Norway: Bergen:** Norwegian Research Council, Norwegian Asthma and Allergy Association, Glaxo Wellcome AS, Norway Research Fund; **Spain:** Fondo de Investigacion Santarias (grant codes 97/0035-01, 99/0034-01 and 99/0034-02), Hospital Universitario de Albacete, Consejeria de Sanidad, **Barcelona:** Sociedad Espanola de Neumología y Cirugía Toracica, Public Health Service (grant code R01 HL62633-01), Fondo de Investigaciones Santarias (grant codes 97/0035-01, 99/0034-01 and 99/0034-02), Consell Interdepartamental de Recerca i Innovació Tecnològica (grant code 1999SGR 00241) Instituto de Salud Carlos III; Red de Centros de Epidemiología y Salud Pública, C03/09, Red de Bases moleculares y fisiológicas de las Enfermedades Respiratorias, C03/011, and Red de Grupos Infancia y Medio Ambiente G03/176, **Huelva:** Fondo de Investigaciones Santarias (grant codes 97/0035-01, 99/0034-01 and 99/0034-02), **Galdakao:** Basque Health Department, **Oviedo:** Fondo de Investigaciones Sanitaria (97/0035-02, 97/0035, 99/0034-01, 99/0034-02, 99/0034-04, 99/0034-06, 99/350, 99/0034-07), European Commission (EU-PEAL PL01237), Generalitat de Catalunya (CIRIT 1999 SGR 00214), Hospital Universitario de Albacete, Sociedad Española de Neumología y Cirugía Torácica (SEPAR R01 HL62633-01), Red de Centros de Epidemiología y Salud Pública (C03/09), Red de Bases moleculares y fisiológicas de las Enfermedades Respiratorias (C03/011) and Red de Grupos Infancia y Medio Ambiente (G03/176; 97/0035-01, 99/0034-01 and 99/0034-02); **Sweden: Göteborg,**

Umea, Uppsala: Swedish Heart Lung Foundation, Swedish Foundation for Health Care Sciences and Allergy Research, Swedish Asthma and Allergy Foundation, Swedish Cancer and Allergy Foundation, Swedish Council for Working Life and Social Research (FAS); **Switzerland: Basel:** Swiss National Science Foundation, Swiss Federal Office for Education and Science, Swiss National Accident Insurance Fund; **UK: Ipswich and Norwich**: Asthma UK (formerly known as National Asthma Campaign).

Cordination: The coordination of this work was supported by the European Commission, as part of their Quality of Life programme (Grant code: QLK4-CT-1999-01237).

ECRHS III

Principal Investigators and senior scientific teams: **Australia: Melbourne** (M. Abramson, G. Benke, S. Dharmage, B. Thompson, S. Kaushik, M. Matheson. **Belgium: South Antwerp & Antwerp City** (J. Weyler, H. Bentouhami, V. Nelen). **Estonia: Tartu** (R. Jõgi, H. Orru). **France: Bordeaux** (C. Raherison, P.O. Girodet) **Grenoble** (I. Pin, V. Siroux, J. Ferran, J.L. Cracowski) **Montpellier** (P. Demoly, A. Bourdin, I. Vachier) **Paris** (B. Leynaert, D. Soussan, D. Courbon, C. Neukirch, L. Alavoine, X. Duval, I. Poirier). **Germany: Erfurt** (J. Heinrich, E. Becker, G. Woelke, O. Manuwald) **Hamburg** (H. Magnussen, D. Nowak, A-M. Kirsten). **Iceland: Reykjavik** (T. Gislason, B. Benediktsdottir, D. Gislason, E.S. Arnardottir, M. Clausen, G. Gudmundsson, L. Gudmundsdottir, H. Palsdottir, K. Olafsdottir, S. Sigmundsdottir, K. Bara-Jörundsdottir). **Italy**: **Pavia** (I .Cerveri, A.Corsico, A. Grosso, F. Albicini, E. Gini, E.M. Di Vincenzo, V. Ronzoni, S. Villani, F. Campanella, M. Gnesi, F. Manzoni, L. Rossi, O. Ferraro) **Turin** (M. Bugiani, R. Bono, P. Piccioni, R. Tassinari, V. Bellisario, G. Trucco) **Verona** (R. de Marco†, S. Accordini, L. Calciano, L. Cazzoletti, M. Ferrari, A.M. Fratta Pasini, F. Locatelli, P. Marchetti, A. Marcon, E. Montoli, G. Nguyen, M. Olivieri, C. Papadopoulou, C. Posenato, G. Pesce, P. Vallerio, G. Verlato, E. Zanolin). **Norway:** (C. Svanes, E. Omenaas, A. Johannessen, T. Skorge, F. Gomez Real). **Spain: Albacete** (J. Martinez-Moratalla Rovira, E. Almar, A. Mateos, S. García, A. Núñez, P. López, R. Sánchez, E. Mancebo), **Barcelona** (J-M. Antó, J.P. Zock, J. Garcia-Aymerich, M. Kogevinas, X. Basagaña, A.E. Carsin, F. Burgos, C. Sanjuas, S. Guerra, B. Jacquemin, P. Davdand, **Galdakao** (N. Muñozguren, I. Urrutia, U. Aguirre, S. Pascual), **Huelva** (J. Antonio Maldonado, A. Pereira, J. Luis Sánchez, L. Palacios), **Oviedo** (F. Payo, I. Huerta, N. Sánchez, M. Fernández, B. Robles). **Sweden: Göteborg** (K. Torén, M. Holm, J-L. Kim, A-C. Olin, A. Dahlman-Höglund), **Umea** (B. Forsberg, L. Braback, L. Modig, B. Järvholm, H. Bertilsson, K.A Franklin, C Wahlgreen), **Uppsala:** (B Andersson, D Norback, U Spetz Nystrom, G. Wieslander, G.M. Bodinaa Lund, K Nisser); **Switzerland: Basel** (N.M. Probst-Hensch, N. Künzli, D. Stolz, C. Schindler, T. Rochat, J.M. Gaspoz, E. Zemp Stutz, M. Adam, C. Autenrieth, I. Curjuric, J. Dratva, A. Di Pasquale, R. Ducret-Stich, E. Fischer, L. Grize, A. Hensel, D. Keidel, A. Kumar, M. Imboden, N. Maire, A. Mehta, H. Phuleria, M. Ragettli, M. Ritter, E. Schaffner, G.A. Thun, A. Ineichen, T. Schikowski, M. Tarantino, M. Tsai). **UK: London** (P. Burney, D. Jarvis, S. Kapur, R. Newson, J. Potts), **Ipswich** (N. Innes), **Norwich** (A. Wilson).

Financial Support: **Australia**: National Health & Medical Research Council. **Belgium: Antwerp South, Antwerp City:** Research Foundation Flanders (FWO), grant code G.0.410.08.N.10 (both sites). **Estonia: Tartu:** SF0180060s09 from the Estonian Ministry of Education. **France:** (**All**) Ministère de la Santé, Programme Hospitalier de Recherche Clinique (PHRC) national 2010. **Bordeaux:** INSERM U897 Université Bordeaux segalen, **Grenoble:** Comite Scientifique AGIRadom 2011. **Paris:** Agence Nationale de la Santé, Région Ile de France, domaine d’intérêt majeur (DIM). **Germany: Erfurt:** German Research Foundation HE 3294/10-1 **Hamburg:** German Research Foundation MA 711/6-1, NO 262/7-1. **Iceland: Reykjavik**: The Landspitali University Hospital Research Fund, University of Iceland Research Fund, ResMed Foundation, California, USA, Orkuveita Reykjavikur

(Geothermal plant), Vegagerðin (The Icelandic Road Administration (ICERA). **Italy:** All Italian centres were funded by the Italian Ministry of Health, Chiesi Farmaceutici SpA, in addition **Verona** was funded by Cariverona foundation, Education Ministry (MIUR). **Norway:** Norwegian Research council grant no 214123, Western Norway Regional Health Authorities grant no 911631, Bergen Medical Research Foundation. **Spain:** Fondo de Investigación Sanitaria (PS09/02457, PS09/00716, 09/01511, PS09/02185, PS09/03190), Servicio Andaluz de Salud, Sociedad Española de Neumología y Cirurgía Torácica (SEPAR 1001/2010), Fondo de Investigación Sanitaria (PS09/02457), **Barcelona:** Fondo de Investigación Sanitaria (FIS PS09/00716), **Galdakao:** Fondo de Investigación Sanitaria (FIS 09/01511) **Huelva**: Fondo de Investigación Sanitaria (FIS PS09/02185) and Servicio Andaluz de Salud **Oviedo:** Fondo de Investigación Sanitaria (FIS PS09/03190). **Sweden:** All centres were funded by The Swedish Heart and Lung Foundation, The Swedish Asthma and Allergy Association, The Swedish Association against Lung and Heart Disease, Swedish Research Council for health, working life and welfare (FORTE) **Göteborg:** Also received further funding from the Swedish Council for Working life and Social Research **Umea:** also received funding from Vasterbotten Country Council ALF grant. **Switzerland:** The Swiss National Science Foundation (grants no 33CSCO-134276/1, 33CSCO-108796, 3247BO-104283, 3247BO-104288, 3247BO-104284, 3247-065896, 3100-059302, 3200-052720, 3200-042532, 4026-028099), The Federal office for forest, environment and landscape, The Federal Office of Public Health, The Federal Office of Roads and Transport, the canton's government of Aargan, Basel-Stadt, Basel-Land, Geneva, Luzern, Ticino, Valais and Zürich, the Swiss Lung League, the canton's Lung League of Basel Stadt/ Basel, Landschaft, Geneva, Ticino, Valais and Zurich, SUVA, Freiwillige Akademische Gesellschaft, UBS Wealth Foundation, Talecris Biotherapeutics GmbH, Abbott Diagnostics, European Commission 018996 (GABRIEL), Wellcome Trust WT 084703MA. **UK:** Medical Research Council (Grant Number 92091). Support also provided by the National Institute for Health Research through the Primary Care Research Network.

Coordination: The coordination was funded through the Medical Research Council (Grant Number 92091).

† deceased

SAPALDIA STUDY

CURRENT SAPALDIA TEAM

Study directorate: N.M. Probst-Hensch (PI; e/g); T. Rochat (p), C. Schindler (s), N. Künzli (e/exp), J.M. Gaspoz (c)

Scientific team: J.C. Barthélémy (c), W. Berger (g), R. Bettschart (p), A. Bircher (a), C. Brombach (n), P.O. Bridevaux (p), L. Burdet (p), D. Felber Dietrich (e), M. Frey (p), U. Frey (pd), M.W. Gerbase (p), D. Gold (e), E. de Groot (c), W. Karrer (p), F. Kronenberg (g), B. Martin (pa), A. Mehta (e), D. Miedinger (o), M. Pons (p), F. Roche (c), T. Rothe (p), P. Schmid-Grendelmeyer (a), D. Stolz (p), A. Schmidt-Trucksäss (pa), J. Schwartz (e), A. Turk (p), A. von Eckardstein (cc), E. Zemp Stutz (e).

Scientific team at coordinating centers: M. Adam (e), I. Aguilera (exp), A. Beckmeyer-Borowko (e), S. Brunner (s), D. Carballo (c), S. Caviezel (pa), I. Curjuric (e), A. Di Pascale (s), J. Dratva (e), R. Ducret (s), E. Dupuis Lozeron (s), M. Eeftens (exp), I. Eze (e), E. Fischer (g), M. Foraster (e), M. Germond (s), L. Grize (s), S. Hansen (e), A. Hensel (s), M.

Imboden (g), A. Ineichen (exp), A. Jeong (g), D. Keidel (s), A. Kumar (g), N. Maire (s), A. Mehta (e), R. Meier (exp), E. Schaffner (s), T. Schikowski (e), M. Tsai (exp)
(a) allergology, (c) cardiology, (cc) clinical chemistry, (e) epidemiology, (exp) exposure, (g) genetic and molecular biology, (m) meteorology, (n) nutrition, (o) occupational health, (p) pneumology, (pa) physical activity, (pd) pediatrics, (s) statistics

Research support: The Swiss National Science Foundation (grants no 33CS30-148470/1&2, 33CSCO-134276/1, 33CSCO-108796, 324730_135673, 3247BO-104283, 3247BO-104288, 3247BO-104284, 3247-065896, 3100-059302, 3200-052720, 3200-042532, 4026-028099, PMPDP3_129021/1, PMPDP3_141671/1), the Federal Office for the Environment, the Federal Office of Public Health, the Federal Office of Roads and Transport, the canton's government of Aargau, Basel-Stadt, Basel-Land, Geneva, Luzern, Ticino, Valais, and Zürich, the Swiss Lung League, the canton's Lung League of Basel Stadt/ Basel Landschaft, Geneva, Ticino, Valais, Graubünden and Zurich, Stiftung ehemals Bündner Heilstätten, SUVA, Freiwillige Akademische Gesellschaft, UBS Wealth Foundation, Talecris Biotherapeutics GmbH, Abbott Diagnostics, European Commission 018996 (GABRIEL), Wellcome Trust WT 084703MA, Exposomics EC FP7 grant (Grant agreement No: 308610).

Acknowledgements: The study could not have been done without the help of the study participants, technical and administrative support and the medical teams and field workers at the local study sites. Local fieldworkers: Aarau: S. Brun, G. Giger, M. Sperisen, M. Stahel, Basel: C. Bürli, C. Dahler, N. Oertli, I. Harreh, F. Karrer, G. Novicic, N. Wyttenbacher, Davos: A. Saner, P. Senn, R. Winzeler, Geneva: F. Bonfils, B. Blicharz, C. Landolt, J. Rochat, Lugano: S. Boccia, E. Gehrig, M.T. Mandia, G. Solari, B. Viscardi, Montana: A.P. Bieri, C. Darioly, M. Maire, Payerne: F. Ding, P. Danieli, A. Vonnez, Wald: D. Bodmer, E. Hochstrasser, R. Kunz, C. Meier, J. Rakic, U. Schafroth, A. Walder.

Administrative staff: N. Bauer Ott, C. Gabriel, R. Gutknecht.
